# RESTORING PHYSICAL FUNCTION THROUGH INTENSIVE VISUAL SIMULATION IN A BALLET DANCER WITH COMPLEX REGIONAL PAIN SYNDROME

**DOI:** 10.2340/jrm.v58.46105

**Published:** 2026-08-03

**Authors:** Lisa STAHLHOFEN, Rob De BIE, Davy LUNEAU, Jens BANSI

**Affiliations:** 1 Department of Neurology, Clinics of Valens, Rehabilitation Center Valens, Valens, Switzerland; 2 Maastricht University, Department of Epidemiology, Care and Public Health Research Institute (CAPHRI), Maastricht, the Netherlands; 3 Dessintey GmbH, Scientific Coordinator, St-Jean-Bonnefonds, France; 4 OST – Eastern Switzerland University of Applied Sciences Department of Health, Switzerland

This case report describes a female ballet dancer in her early forties who started inpatient rehabilitation 10 months after being diagnosed with complex regional pain syndrome (CRPS) type II in her left foot. Using a novel therapeutic approach, in which Intensive Visual Simulation (IVS) is part of the treatment plan, altered the progress of her rehabilitation. The patient presented with known CRPS risk factors, including female sex, extremity surgery, and psychological stress (1). CRPS was diagnosed with the Budapest Diagnostic Criteria, published by the International Association for the Study of Pain (IASP) (2). CRPS occurs in all racial groups (2), with a higher prevalence in females (3). Epidemiological data remain limited. A recent meta-analysis from 2025 reported pooled global prevalence rates of 3.04% at 12 and 6.46% at 24 months (4). In Europe, 5 per 10,000 individuals are affected, with 14–27% continuing to meet the diagnostic criteria after 12 months (5).

CRPS is defined as a chronic pain disorder characterized by pain disproportionate to the inciting event that persists beyond normal tissue healing (1). This is often accompanied by sensory, motor, and autonomic disturbances like allodynia, hyperalgesia, skin discolouration, temperature asymmetry, and sudomotor alterations (5). CRPS is commonly triggered by trauma or surgery and is classified into type I (no confirmed nerve injury) and type II (with nerve injury) (1).

The pathophysiology of CRPS is multifactorial, involving inflammatory processes, peripheral and central sensitization, autonomic dysfunction, and neuroplastic changes (1). Additionally, behavioural factors like fear avoidance and disuse may worsen functional outcomes (6). Higher self-efficacy has been associated with improved long-term function and reduced disability in chronic pain populations (7).

## CASE REPORT

A female ballet dancer in her early forties presented for rehabilitation 10 months after CRPS diagnosis of her left foot. Her medical history included hallux-valgus correction surgery and a hyperextension trauma of the big toe without fracture. Neurophysiological testing indicated possible axonal damage of the tibial and peroneal nerves consistent with her CRPS type II. Five months after diagnosis, she developed severe throbbing pain in her foot. Magnetic resonance imaging (MRI) revealed a stress fracture in the distal part of the fourth metatarsal without preceding trauma. Besides being a ballet dancer, she works as a management consultant with high-performance demands. However, since surgery, she has been on sick leave due to severe CRPS symptoms, including persistent pain and inability to sleep. Her activities of daily living and social participation were markedly reduced.

### Diagnostic assessment and clinical findings

Before admission to the clinic, the Budapest Criteria by IASP were fulfilled (2):

- Continuous disproportionate pain.
- Sensory, vasomotor, sudomotor and motor/tropic symptoms.
- At the time of evaluation, there was vasomotor dysregulation and allodynia.
- No alternative diagnosis.

**Fig. 1:**
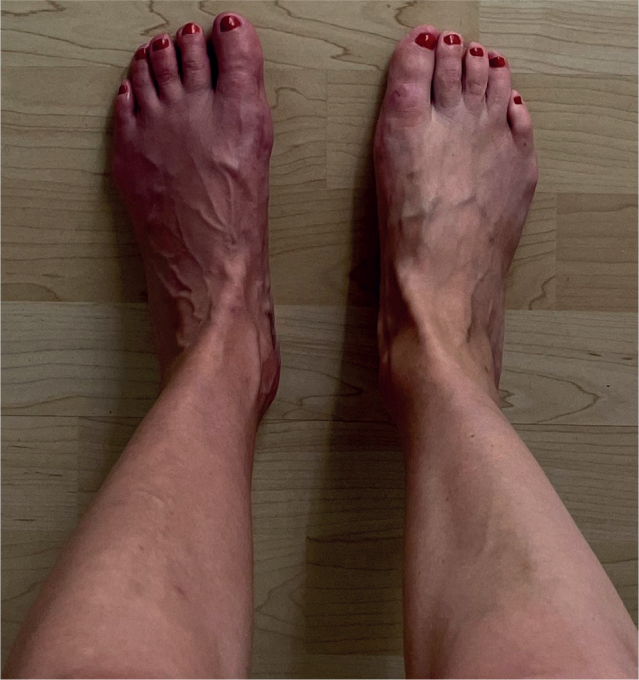
Complex regional pain syndrome (CRPS) presentation in the patient’s left foot before entering clinic.

Symptoms included allodynia, hyperesthesia, skin discoloration (red or blue skin, sometimes accompanied by white spots), oedema, sweating changes, motor weakness, and hair growth with nail changes (Fig. 1).

The patient described sensations as “burning, squeezing, needling, hammering, swelling, colouring of the foot, just to name a few of the common sensations”. Pain spread from the big toe to the entire foot and was most severe at night, causing severe sleep deprivation.

On admission to the clinic:

- Subjective felt pain was scored, NPRS 4–5;
- On the 6-Minute Walk Test (6MWT) she walked 380 m;
- Timed Up and Go (TUG) took her 9 s with a post-op shoe;
- 10-Meter Walk Test (10MWT) took her 8 s and 16 steps;
- The Single-Leg Stance was not possible;
- Patient-Reported Outcomes Measurement Information System (PROMIS-10) Global Physical Health and Global Mental Health were scored as a raw score of 8 and 9, translating to poor T-scores compared with the general population.

### Therapeutic interventions

Prior outpatient rehabilitation included physiotherapy, corticosteroids (opioids) (5), acupuncture, hypnosis, massage, and hyperbaric oxygen therapy without sufficient relief. Due to the long-lasting complaints and non-significant effect of treatment, former healthcare professionals considered the prognosis to be poor.

Inpatient rehabilitation combined active standard therapies like yoga, aquatic therapy, resistance, and endurance training with IVS. Evidence suggests that combining graded motor imagery and mirror therapy improves CRPS outcomes (8). IVS integrates motor imagery, mirror therapy, and action observation to induce cortical sensorimotor activation, thereby promoting relearning (8). The IVS4 device enables IVS therapy for the lower limbs.

Initial IVS sessions involved mirrored recordings of the unaffected foot performing basic movements like toe and heel raises, and lifting of the foot with tilting the foot forward and backwards. Early sessions on the IVS4 triggered symptom exacerbations, including discolouration and pain. The patient indicated that putting the foot on the ground was particularly painful, although she was only observing the foot movements and not actively performing them. Therefore, sessions were shortened to 5 min and gradually adjusted. This allowed the patient to adapt to the device and the movement observation of her affected foot.

**Fig. 2:**
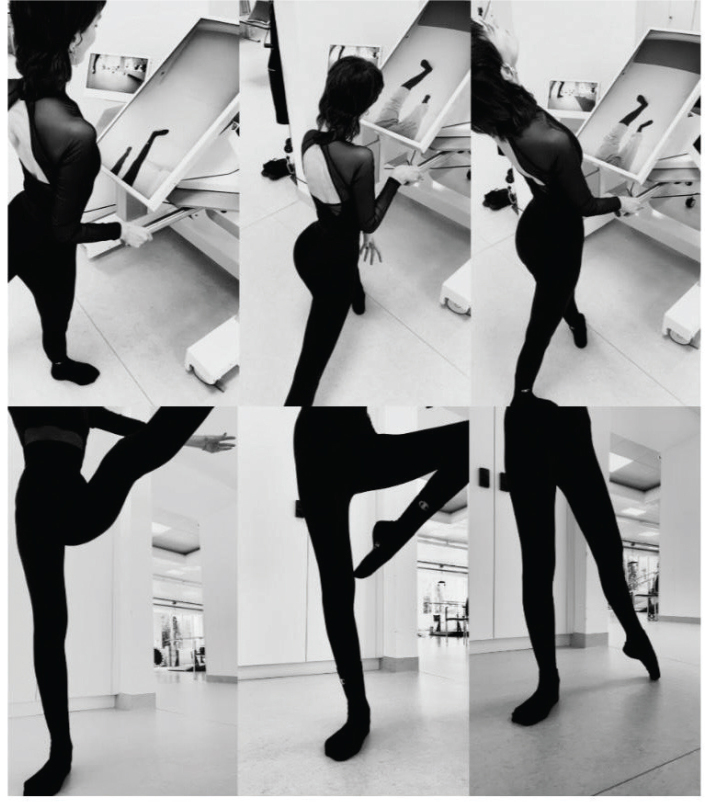
Ballet exercise mirrored during Intensive Visual Simulation on the IVS4.

A key therapeutic modification was the integration of ballet-specific movements. Exercises like *Relevés*, *Pliés*, and *Tendus* were introduced and accompanied by ballet music (Fig. 2). These familiar movements improved emotional engagement and reduced negative responses. Over time, exercise complexity increased without exacerbation of symptoms. After 2 weeks of IVS treatment, the patient began actively performing movements during IVS. Sessions increased to 30 mins, performed 2–3 times daily. IVS was combined with physiotherapy focusing on graded exposure and stability training to enhance self-efficacy.

The patient reported the effects of the IVS treatment as follows: “It is a positive stimulus to my brain. Seeing my foot performing a ballet movement is highly emotional and a big motivator to continue IVS practice. Pain decreased, and CRPS symptoms improved.”

The exercises were enhanced to allow for more complex movement sequences, including *Rond de Jambe par Terre* and more advanced combinations of *Tendu & Pliés*, requiring more precise stabilization and higher strength efforts. Time spent on IVS4 significantly increased from 5 min per session to sessions up to 30 min while executing full dancing routines that she performed 2–3 times a day, without pain exacerbations. These IVS exercises were then combined with stability training and graded activity during physiotherapy sessions, aiming to increase self-efficacy and gradual exposure to more activity without worsening pain.

### Follow-up and outcomes

After 15 days of rehabilitation:

- 6MWT increased by 140m (+ 37%).
- 10MWT 6 s (25%) and 13 steps (+19%).
- MiniBEST feasible – all items were successfully performed, including the execution of a single-leg stance.

At discharge:

- 6MWT increased by 220m (+57%) compared with admission.
- Health-related quality of life, measured with the PROMIS-10, improved to a score of 15 on Global Physical Health and 18 on the Global Mental Health.
- NPRS reduced to 1.

An overview of all relevant test scores is presented in Table I.

**Table I:** Test scores over time

Test	Clinic entry	After 15 days	Clinic exit (after 6 weeks)
NPRS	4–5	2–3	1
6MWT	380 m	520 m	600 m
10MWT> Time> Steps	8 s16 steps	6 s13 steps	7 s13 steps
TUG	9 s	6 s	5 s
PROMIS-10			
> Global Physical Health	8*		15*
> Global Mental Health	9*		18*

NPRS: Numeric Pain Rating Scale; 6MWT: 6-minute walk test; 10MW: 10-meter walk test; TUG: Timed Up and Go; PROMIS-10: Patient Reported Outcome Measures Information System; s: seconds.

* Raw score of PROMIS-10.

## DISCUSSION

This case demonstrates that IVS combined with patient-specific meaningful activities improved physical function and reduced pain in CRPS. The NPRS improvement exceeded a minimal clinically important difference of 4 points, and the functional gains in the 6MWT and TUG both indicate clinically relevant improvement.

CRPS requires individualized, multidisciplinary disease management. However, to date, only one guideline on CRPS treatment management is available, published by the UK Royal College of Physicians (RCP) (1). Its treatment aims include pain reduction, functional restoration, and improved quality of life (1). In this case, balancing activity progression with symptom control was critical due to the patient’s high-performance mindset. The intervention enhanced self-efficacy by addressing fear-avoidance behaviour through graded exposure and meaningful activity integration. This aligns with evidence that links self-efficacy to improved outcomes in chronic pain (9). The integration of ballet into the training programme addressed both psychological and identity-related factors. Positive emotional engagement likely contributed to symptom modulation, consistent with the gate control theory of pain (10). IVS enabled graded re-exposure to movement without physical overload.

Although mirror therapy and motor imaginary are established therapies, evidence for IVS in CRPS is lacking. This case suggests that the combination of IVS with ballet dancing exercises enhances engagement and adherence by allowing personalized, meaningful movement observation. Regular exercising resulted in increased PROMIS-10 scores that indicate improvement in health-related quality of life. Moreover, the gain in confidence allowed an increase of the active therapies into her daily schedule. The increase in the patient’s self-efficacy highlights the potential of IVS to enhance physical function while reducing avoidance coping strategies.

## CONCLUSION

Intensive Visual Simulation combined with patient-specific activities like ballet improve pain, physical function, and self-efficacy in complex regional pain syndrome. This approach shows promising results as an adjunct to multidisciplinary rehabilitation and warrants further investigation.

### Patient perspective

“CRPS was the most devastating, frightening and challenging experience in my life. The constant pain, inability to move or sleep and the loss of my identity as a dancer were overwhelming. Recovery required persistence, patience and trust. IVS helped me to reconnect with movement and to regain hope.”
